# A Case of Absence of All Sensation

**Published:** 1919

**Authors:** 


***A Case of Absence of all Sensation.*** By E.D. Roberts. *The Lancet*, October 19, 1918.

The remarkable case described below enlisted in the Canadian Army in 1916. In June, 1918, he was admitted to the Military Isolation Hospital, Aldershot, for an attack of mumps, and it was then that the following observations were made :

The patient was a well-developed, powerful-looking man. Mentally, he was perfectly sound, rather above than below the average level of intelligence. As far as history goes, he would appear to be alone in presenting the peculiar characteristics here described. As a child he appears to have possessed a slight degree of cutaneous sensibility, and he dates its complete absence from the time when he had an attack of yellow fever at the age of 17. This was his only previous malady, and he has always enjoyed robust health.

There is complete absence of both superficial and deep tactile sense over the whole skin surface, and also over mucous membrane. There is no consciousness of deep vibration. He does not feel the ground with his feet, and experiences difficulty when walking at night.

The sense of pain is completely absent. In 1916 he underwent the operation of double inguinal hernia without anesthetic of any kind. He never suffers from headache, toothache, abdominal or other visceral pain of any description. The cornea shares in general anesthesia.

Thermal sensation is also absent. There is no perception of temperature in food or drink. There is insensibility to atmospheric changes of temperature.

The muscular sense and sense of position seem to be completely absent. With the eyes closed, if asked to make any movement with his arms, he is incapable of doing so, saying that he cannot tell whether his arms are moving or not. On the other hand, when standing upright, with closed eyes, if he is told to walk towards the observer, he does so without trouble. If any small object is placed in one hand and a much heavier one in the other, he is incapable of saying which hand holds the heavier weight. He appears ignorant of the meaning of fatigue.

The sense of position is equally absent. With visual control all his movements are perfectly co-ordinated and in every way normal.

He exhibits the phenomenon of fixation of the limbs in the positions in which they are placed.

The sense of taste is completely absent. He eats anything that takes his fancy. It appears that he is never conscious of hunger or thirst.

The sense of smell is practically non-existent.

The bowels usually act regularly once a day. There is no conscious desire to defecate beyond what he describes as an occasional " rolling " in the abdomen.

Micturition ordinarily occurs during the action of the bowels once in 24 hours. There is never a desire to micturate.

Finally, he seems to be without most of the common emotions.

Diagnosis of syringomyelia combined with hysteria is suggested as one which seems to meet the case.

